# Characterization of bony anatomic regions in pediatric and adult healthy volunteers using diffuse optical spectroscopic imaging

**DOI:** 10.1117/1.JBO.25.8.086002

**Published:** 2020-08-12

**Authors:** Hannah M. Peterson, Anup Tank, David S. Geller, Rui Yang, Richard Gorlick, Bang H. Hoang, Darren Roblyer

**Affiliations:** a Boston University, Department of Biomedical Engineering, Boston, Massachusetts, United States; b Montefiore Medical Center, Department of Orthopaedic Surgery, Bronx, New York, United States; c MD Anderson Cancer Center, Division of Pediatrics, Houston, Texas, United States

**Keywords:** *in vivo* imaging, near-infrared, diffuse optics, tissue spectroscopy, bone, translational research, pediatrics, sex differences

## Abstract

**Significance:** Diffuse optical spectroscopic imaging (DOSI) measures quantitative functional and molecular information in thick tissue in a noninvasive manner using near-infrared light. DOSI may be useful for diagnosis and prognosis of bone pathologies including osteosarcoma and Ewing’s sarcoma, but little is currently known about DOSI-derived parameters in bony anatomic locations where this disease occurs.

**Aim:** Our goal is to quantify the optical characteristics and chromophore content of bony anatomic locations of healthy volunteers and assess differences due to anatomic region, age, sex, ethnicity, race, and body fat.

**Approach:** Fifty-five healthy volunteers aged 4 to 72 were enrolled in the study. The optical properties and quantitative tissue concentrations of oxyhemoglobin, deoxyhemoglobin, water, and lipids were assessed at the distal humerus, distal femur, and proximal tibia. Body fat was assessed using skinfold calipers. One volunteer underwent a more comprehensive body scan from neck to foot to explore chromophore distributions within an individual. Regression analysis was used to identify the most important sources of variation in the measured data set.

**Results:** Statistical differences between bony locations were found for scattering, water, and lipids, but not for hemoglobin. All chromophores had statistical differences with sex, but there were no significant age-related correlations. Regression analysis revealed that body fat measured with skinfold calipers was the most important predictor of oxy-, deoxy-, total hemoglobin, and lipids. Hemoglobin and lipid levels were highly correlated (ρ≥0.7) over the subject population and within the single-subject body scan.

**Conclusions:** DOSI can successfully measure bony anatomic sites where osteosarcomas and Ewing’s sarcomas commonly occur. Future studies of bone pathology using DOSI should account for the variation caused by anatomic region, sex, race and ethnicity, and body fat as these cause substantial variations in DOSI-derived metrics.

## 1 Introduction

The blood supply, metabolism, and composition of bone are known to be altered in a variety of pathologies of the skeletal system including osteoporosis, diabetes, anemias, and some forms of arthritis.^1^^,^^2^ Likewise, sarcomas, malignant tumors of bone and connective tissue are known to have substantially altered blood supply at the tumor site, often with hypoxia and necrosis.^3^^,^^4^ The quantitative measurement of bony regions of the body could assist in diagnosis, prognosis, and basic understanding of the pathophysiology of these diseases.

The hemodynamic properties of bone present a challenge for measurement for many standard-of-care imaging modalities including positron emission tomography and contrast-enhanced magnetic resonance imaging due to its high density and mineral content.^2^ Near-infrared light (600 to 1000 nm), however, is able to penetrate bony regions due in part to the relatively low absorption of hemoglobin, water, lipids, and collagen in this wavelength range. Diffuse optical spectroscopic imaging (DOSI) is a noninvasive near-infrared imaging technique that measures quantitative concentrations of functional hemodynamic and molecular species including oxyhemoglobin (${\mathrm{HbO}}_{2}$), deoxyhemoglobin (HHb), water, and lipids. DOSI is label-free and can measure tissue up to several centimeters deep, making it a powerful technique for measuring deep tissue physiology.^5^ DOSI has been used extensively in the context of breast cancer^6^^–^^9^ and exercise physiology^10^ among others. The use of DOSI and similar techniques for earlier detection, prevention, and monitoring of bone pathologies is more recent, and the variation among anatomic sites, sex, and age is only beginning to be characterized.^2^^,^^11^^–^^13^

We recently evaluated the feasibility of using DOSI to measure sarcomas in pediatric patients for the first time.^13^ Bone and soft tissue sarcomas comprise 12% of all childhood cancers and 5-year disease-free survival and overall survival for localized bone sarcomas have remained at ∼70% for the last 40 years, representing a major clinical need.^14^^,^^15^ The optical characterization of sarcomas may provide a new means to diagnose and track treatment efficacy in this patient population.

The goal of this study was to quantify the optical properties and functional hemodynamic and molecular content of bony regions where sarcomas typically occur in adult and pediatric healthy volunteers using DOSI. This would provide a baseline of comparison for DOSI measurements taken in sarcoma patients. This study focused on three anatomic locations in healthy volunteers: the distal humerus, distal femur, and proximal tibia. These sites account for 66% of osteosarcoma locations.^16^ The average DOSI derived values at the medial distal humerus, distal femur, and proximal tibia are presented in the context of age, sex, race, ethnicity, and body fat. Correlations between DOSI metrics and body fat are also presented as well as an evaluation of spatial variations of chromophore concentrations over the body.

## 2 Methods

### 2.1 DOSI Instrumentation

A custom benchtop DOSI system was used to acquire both frequency-domain and broadband continuous wave measurements. A handheld probe delivered amplitude modulated near-infrared (NIR) light and broadband light to the tissue via 400-μm and 1-mm optical fibers, respectively. Frequency-domain measurements were acquired using six lasers (658, 690, 785, 808, 830, and 850 nm, part numbers: HL6501MG, HL6738MG, L785P100, L808P030, HL8338MG, and L850P030, respectively, all lasers were acquired from Thorlabs, Newton, New Jersey, USA). These lasers are rated for maximal output powers between 30 and 90 mW, but light levels at tissue were substantially lower due to the use of submaximal operating currents and fiber coupling losses. Light levels from the system were delivered to the tissue with a series of 400-μM optical fibers and optical power at the tissue surface was within American National Standards Institute (ANSI) limits for skin exposure. Laser light was amplitude modulated in a sweep from 50 to 500 MHz and was detected by a C5658 detector module customized by the manufacturer with a 1-mm active area APD (S6045-03; both module and APD are from Hamamatsu Photonics, Hamamatsu, Japan). A commercial network analyzer (E5061B 100 kHz to 1.5 GHz ENA Series Network Analyzer, Agilent Technologies, Santa Clara, California, USA) was used to generate rf and measure detected amplitude and phase. Broadband light from a tungsten-halogen lamp was detected by an NIR spectrometer from 600 to 1000 nm (AvaSpec HS2040XL with a 200-μm slit, 600  lines/mm grating, and 10 to 12 nm FWHM spectral resolution, Avantes Inc., Broomfield, Colorado, USA). A custom LabVIEW (National Instruments, Austin, Texas) code was developed to control the system. The handheld probe was the same as used by Peterson et al.^13^ All measurements for this study were taken with a source–detector separation of 28 mm.

### 2.2 Volunteer Eligibility and Enrollment

Any healthy volunteer was eligible as a participant for this study. Informed consent was obtained from all volunteers. Minors (<18 years) gave assent to the best of their ability to understand and their parent or guardian gave written informed consent. This project was approved by the Institutional Review Board at Boston University. The target sample size was 55 volunteers, which is comparable to other similar studies which used the same^17^ or smaller^11^^,^^12^ sample sizes.

### 2.3 Estimates of Body Fat Percentage

Estimates of body fat percentage were calculated by measurements of subcutaneous tissue using the Lange Skinfold Caliper (Beta Technology, Santa Cruz, California). Skinfold measurements were taken at the biceps, triceps, subscapular, and the suprailiac. The sum of the four skinfold measurements was converted to the equivalent fat content as a percentage of body weight using the Lange Skinfold Caliper Operators Manual.^18^ For volunteers <18 years, only triceps and subscapular skinfold measurements were taken and converted to a percentile compared to the national average. The percentile value reports the percentage of the national sample who had or exceeded that skinfold thickness, e.g., a high percentile value reflects a low body fat percentage.

### 2.4 Imaging Procedures and Data Processing

A DOSI measurement procedure developed for bony measurements used in our prior published study was modified for measurements taken in this study.^13^ In this study, measurements were made on the left and right medial distal humerus, distal femur, and proximal tibia.

**Fig. 1:**
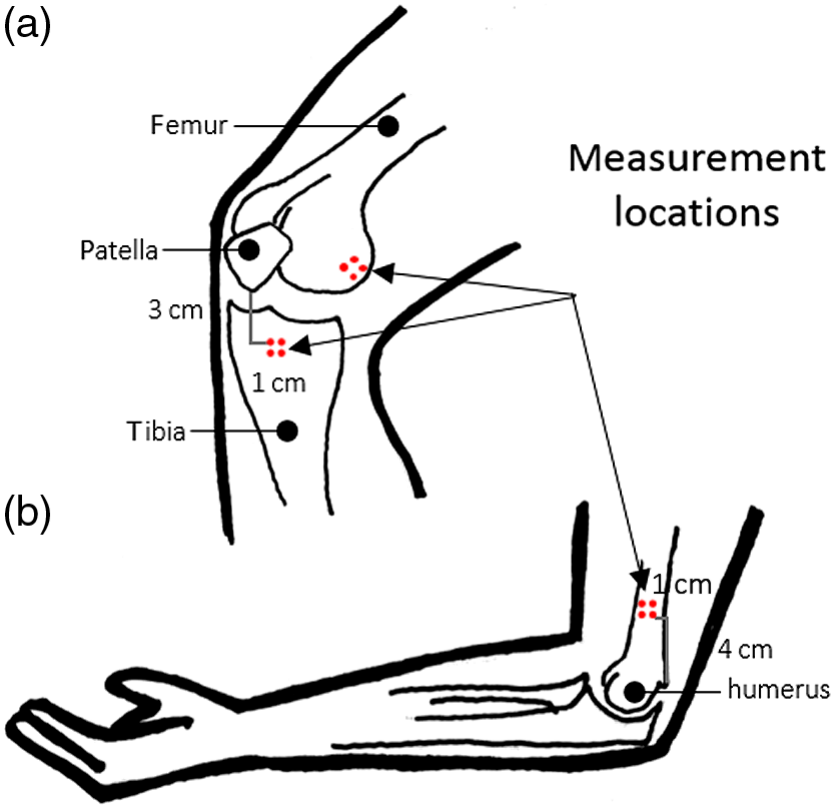
Schematic of DOSI measurement locations: (a) medial epicondyle of the femur and 3 cm from the distal end of the patella and (b) 4 cm from the medial epicondyle of the humerus.

The corners of a $1\text{-}{\mathrm{cm}}^{2}$ grid were transferred onto the skin surface at each location using a transparency and nonpermanent surgical marker. As shown in Fig. 1, all volunteers were measured 4 cm from the medial epicondyle of the humerus, on the medial epicondyle of the femur, and 3 cm from the distal end of the patella on the right and left sides of the body. The transparency included distinctive features to serve as a reference, e.g., freckles, moles, scars, and bone landmarks. The landmarks helped to identify the same region of interest for any subsequent measurements.

An operator scanned the handheld probe over the measurement square, taking a measurement at each point. Care was taken to apply as little pressure as possible while maintaining contact to avoid deforming the tissue of interest. Each volunteer in this study had a total of eight distinct measurements on the distal humerus, the distal femur, and the proximal tibia—a total of 24 point measurements. All measurements for this study were taken with a source–detector separation of 28 mm.

Absorption and reduced scattering coefficients ($\mu_{a}$ and $\mu_{s}^{\prime }$, respectively) were calculated from simultaneously fitting calibrated frequency-domain amplitude and phase measurements from 50 to 400 MHz to a P1 diffusion approximation of the radiative transport equation, assuming a homogenous, semi-infinite media.^5^^,^^19^ A power law fit ($\mu_{s}^{\prime }=a\lambda^{b}$) of the reduced scattering coeffients was used to calculate the a and b parameters normalized to 800 nm. Broadband absorption from 650 to 1000 nm was fit and scaled to the frequency-domain absorption measurements.^5^^,^^20^^,^^21^ Quantitative concentrations of oxyhemoglobin, deoxyhemoglobin, water, and lipids were calculated using Beer’s law from broadband absorption spectra and known extinction coefficients.^22^^–^^25^ Total hemoglobin concentration (THC) and tissue oxygen saturation (${\mathrm{StO}}_{2}$) were calculated from oxyhemoglobin and deoxyhemoglobin concentrations.

### 2.5 Data Quality Control

Several quality control measures were taken to ensure high-quality data. The signal-to-noise ratio (SNR) for each laser was calculated as the raw amplitude divided by a dark measurement. The dark measurement was calculated as the average of five measurements taken on a highly absorbing black phantom. The SNR was calculated for all measurements and averaged as a function of the modulation frequency. For a laser to be included in the data set for a volunteer, the 90th percentile of the SNR had to be >4.

Light leakage from poor contact from measurements on angular and bony surfaces was detected using the presence of ambient fluorescent light peak at 612 nm. Measurements were excluded if there was a peak between 609 and 615 nm in the raw continuous wave measurement. Measurements were also excluded if the continuous wave measurement was not greater than the the noise floor or if the measurement was saturated.

After data processing using the methods described in Sec. 2.4, the broadband reduced scattering coefficients were fit to a power law and checked to ensure they produced a negative slope, and chromophore values were checked to ensure they were within a physiological range (i.e., lipid and water percentage cannot exceed 100%). Data that did not meet these requirements were excluded. Any points over tattoos were also excluded.

### 2.6 Comparison of Optical Properties and Chromophore Concentrations at Bony Locations

The average optical properties and chromophore values were computed from the left and right sides of the humerus, femur, and tibia for each volunteer. Differences between anatomic sites were evaluated as well as correlations between chromophores, body fat, and age.

The Shapiro–Wilk test was used to evaluate whether data were normally (Gaussian) distributed. For Gaussian distributions, the one-way repeated measures analysis of variance (ANOVA) with Tukey’s honestly significant difference (HSD) *post hoc* test was used to compare differences in chromophores between the humerus, femur, and tibia. For non-Gaussian distributions, differences in chromophores between the bony locations were compared using the Kruskal–Wallis H-test with Dunn’s test for multiple pairwise comparisons.

The Pearson’s correlation coefficient was used to quantify the relationship between DOSI lipid concentrations at a bony location and the body fat percentage. A Student’s T-test was used to compare sex differences within a chromophore at a bony location and no multiple comparison test was used.

All statistics were calculated using SciPy 1.0.0 in Python version 2.7.13.^26^^,^^27^

### 2.7 Quantification of Variation with Regression Analysis

A set of ordinary least squares models were used to identify the independent variables that accounted for variation in DOSI metrics. The ordinary least squares model used is shown as follows:

DOSI metric∼bony location+body fat+sex+age+race+ethnicity+body fat*sex, (1)

where DOSI metric is the measurement of the optical property or chromophore value averaged from left and right sides on a bony location for each volunteer and the independent variables are age, sex, bony location, body fat, race, and ethnicity. An interaction term is also included for sex and body fat percentage (body fat*sex). The reference variables for the categorical variables bony location, sex, race, and ethnicity are, respectively, femur, female, Asian, and Hispanic. Each DOSI metric was modeled using the full model described in Eq. (1) and then reduced one term at a time until only significant variables remained. The coefficients of significant variables and the adjusted $R^{2}$ ($R_{\mathrm{adj}}^{2}$) were recorded. The $R_{\mathrm{adj}}^{2}$ quantifies the proportion of variance described by the model based on the number of observations and the degrees of freedom.

After identifying the significant variables, each significant variable was removed from the reduced model and the $R_{\mathrm{adj}}^{2}$ recorded and subtracted from the reduced model $R_{\mathrm{adj}}^{2}$. The most influential variable for the reduced model is defined as the variable with the largest $\Delta R_{\mathrm{adj}}^{2}$.

## 3 Results

### 3.1 Volunteer Enrollment

Fifty-five volunteers were enrolled in the study. The volunteer characteristics are shown in Table 1. Volunteers are identified by the study number with a seven-digit identifier in the form 3367-000.

**Table 1:** Volunteer characteristics.

Variable	Enrolled
Volunteers (n)	55
Adult (≥18 years)	48 (87%)
Minor (<18 years)	7 (13%)
Age (years)	
Median	27
Range	[4 to 72]
Sex (n)	
Male	25 (45%)
Female	30 (55%)
Race (n)	
White	27 (49%)
Black/African–American	3 (5%)
Asian	17 (31%)
Multiracial	1 (2%)
Other	2 (4%)
Unknown	5 (9%)
Ethnicity (n)	
Hispanic or Latino	7 (13%)
Not Hispanic or Latino	48 (87%)
Adult body fat percentage (n=48)	
Median (%)	21.7
Range (%)	[4.8 to 41.3]
Off the chart (n)	1
Unknown (n)	1
Minor skinfold percentiles^a^ (n=7)	
Median (%)	85
Range (%)	[25 to 99]
Unknown (n)	2

a Percentile value reflects the percentage of boys and girls in the national sample who had or exceeded that skinfold thickness.

**Fig. 2:**
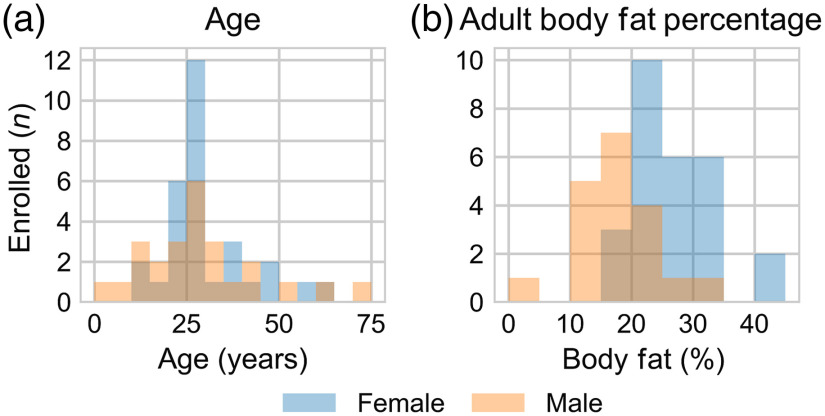
Distribution in (a) age (n=55) and (b) adult body fat percentage of male (orange) and female (blue) volunteers.

Of the 55 volunteers, 7 (13%) were minors (<18 years). The youngest volunteer was 4 years old and the oldest was 72 years old; the distribution of ages is shown in Fig. 2(a). 55% of the volunteers were female (n=30). The distribution of ages between females and males was not statistically different (p=0.82). The volunteers were racially and ethnically diverse; however, the majority were white (n=27, 49%) and not Hispanic or Latino (n=48, 87%).

Table 1 reports body fat percentages for adults (n=48) and national skinfold percentiles for minors (n=7). One adult volunteer and two minors did not participate in a skinfold measurement and their respective body fat percentages and skinfold percentiles are unknown. The exact body fat percentage of another adult volunteer is unknown as the sum of skinfold measurements and age did not match a body fat percentage on the chart. The distribution of adult body fat percentages for males and females is shown in Fig. 2(b). Females had a statistically higher body fat percentage compared to males (p<0.001).

### 3.2 Data Quality Control

Of the 1320 DOSI measurements, 937 (71%) met the quality control procedures outlined in Sec. 2.5.

No volunteers were removed from the data set because of low SNR. However, seven volunteers were processed with five of the six lasers. Five volunteers (1 female and 4 males) were processed without the 658-nm laser due to low SNR and 2 volunteers (1 female and 1 male) were processed without the 830-nm laser due to beam misalignment. On average, 658 nm had the lowest SNR and 785 nm had the highest SNR. SNR was greater in individuals with higher body fat percentage (data not shown).

No volunteers were removed from the data set because of contact issues, although individual measurement points were removed in some cases. Points that did not meet the quality control procedures were rejected for light leakage (n=274, 72% of rejected data), nonphysiological chromophore values, or nonnegative scattering slope fits (n=109, 28% of rejected data).

### 3.3 Optical Properties and Chromophore Concentrations

**Fig. 3:**
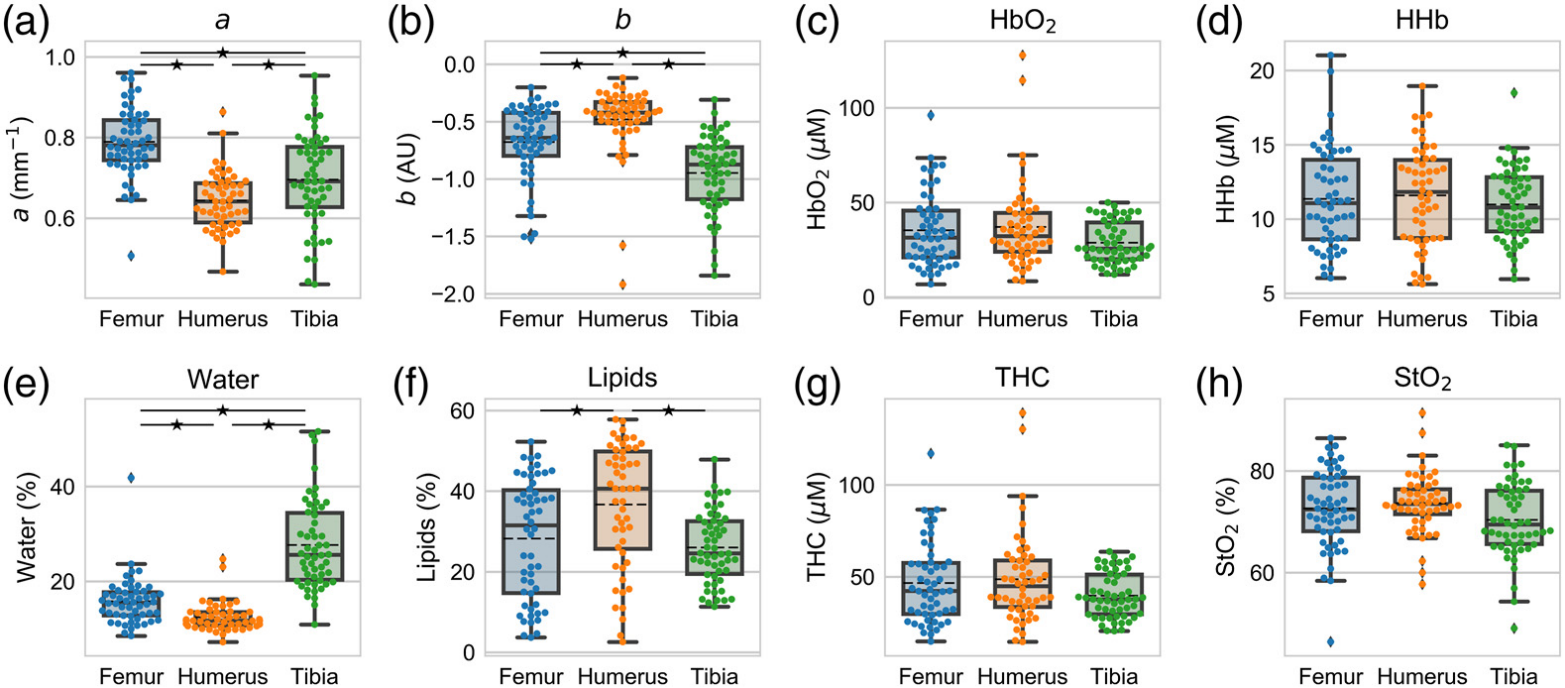
DOSI-derived metrics (a) a parameter, (b) b parameter, (c) ${\mathrm{HbO}}_{2}$, (d) HHb, (e) water, (f) lipids, (g) THC, and (h) ${\mathrm{StO}}_{2}$ for the humerus (orange), femur (blue), and tibia (green). Each point is the average of the left and right measurements for an individual. The box plot shows the mean (dashed black line), median (solid black line), interquartile range (box), and whiskers extending to 1.5 times the quartiles (thin black line). ⋆ represents a statistical significance between bony locations at p=0.05.

The mean and standard deviation of DOSI metrics for each bone are shown in Table 2 and their distributions are shown in Fig. 3.

**Table 2:** Mean and standard deviation for optical properties and chromophore concentrations in three bony anatomic regions.

Variable	Femur	Humerus	Tibia
a (${\mathrm{mm}}^{-1}$)	0.8±0.1	0.6±0.1	0.7±0.1
b (Arbitrary unit)	−0.7±0.4	−0.5±0.3	−1.0±0.3
${\mathrm{HbO}}_{2}$ (μM)	35.3±19.4	37.0±22.4	28.8±11.0
HHb (μM)	11.3±3.4	11.5±3.3	10.9±2.4
Water (%)	16.0±5.0	12.5±3.0	27.8±9.2
Lipids (%)	28.9±15.1	36.9±15.1	26.2±8.7
THC (μM)	46.6±22.1	48.6±24.6	39.8±12.3
${\mathrm{StO}}_{2}$ (%)	72.8±7.9	73.7±5.9	70.5±7.4

The Shapiro–Wilk test identified that the a parameter and tissue oxygen saturation were likely sampled from a Gaussian distribution. The statistical differences between bony regions for these metrics were calculated with the one-way ANOVA with Tukey’s HSD *post hoc* test. The b parameter, oxyhemoglobin, deoxyhemoglobin, lipids, water, and THC were determined to not likely be sampled from a Gaussian distribution (p<0.05 for all). The Kruskal–Wallis H-test with Dunn’s test was used to identify statistical differences between bony regions for these metrics.

Each bony region was statistically different from the others for the a [Fig. 3(a)] and b parameters [Fig. 3(b)] as well as water concentration [Fig. 3(e)]. The lipid concentration of humerus was statistically higher than the concentrations of the femur and tibia (p<0.05); however, the femur and tibia did not statistically differ from each other [Fig. 3(f)]. None of anatomic sites were statistically different from each other for the hemoglobin metrics [p>0.05, Figs. 3(c), 3(d), 3(g), and 3(h)]. The mean values of oxyhemoglobin, deoxyhemoglobin, water, and lipids for the femur, humerus, and tibia were similar with large standard deviations (Table 2 and Fig. 3).

**Fig. 4:**
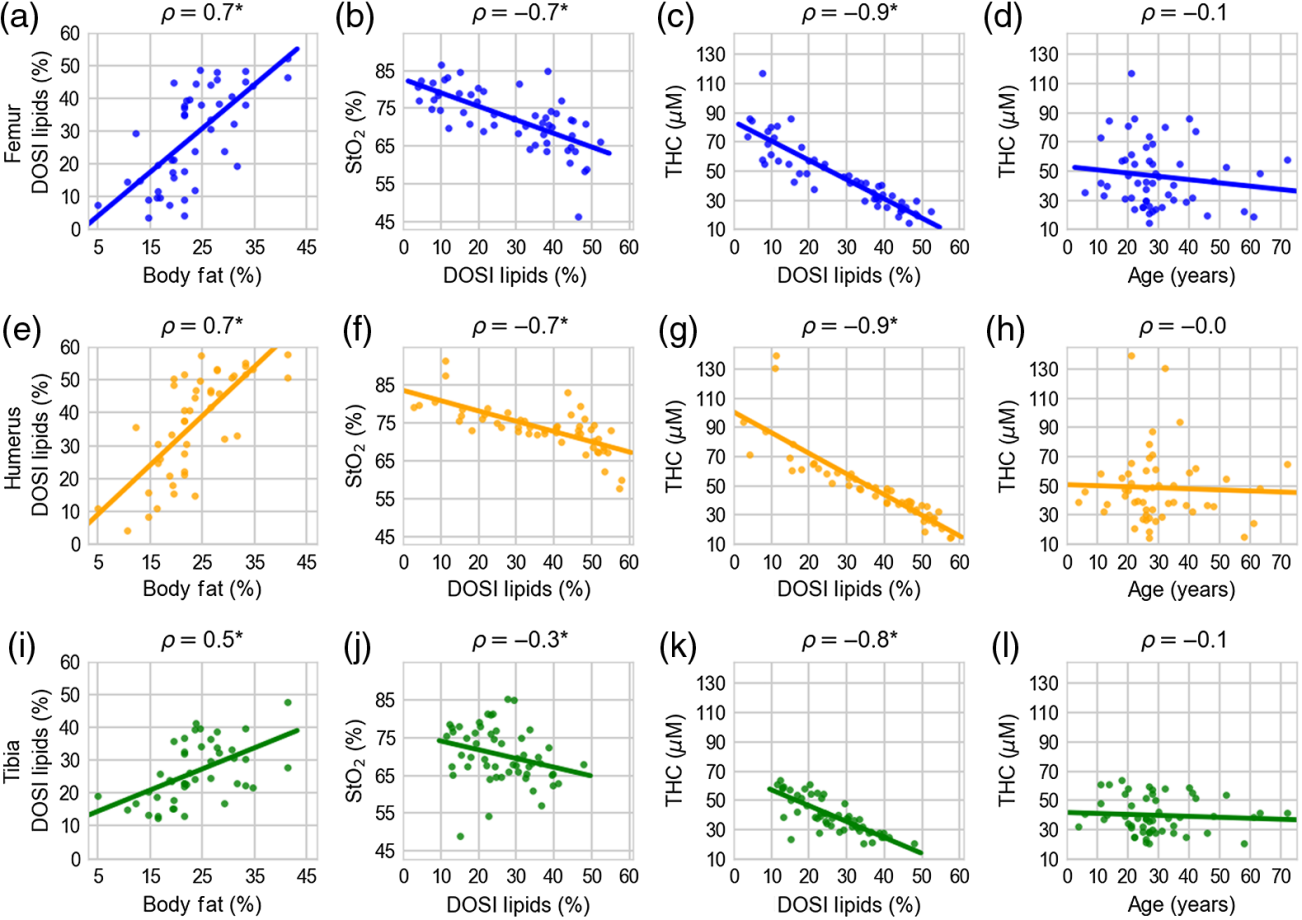
(a), (e), (i) Correlations between adult DOSI lipids and body fat percentage; (b), (f), (j) tissue oxygen saturation and DOSI lipid measurements; (c), (g), (k) THC and DOSI lipid measurements; and (d), (h), (l) THC and age for the femur (blue), humerus (orange), and tibia (green). Each point is the measurement for an individual and the line is the best fit from a linear regression. ⋆ represents statistical significance at a significance level of p=0.05

Several significant correlations were identified both between DOSI-derived metrics as well as between DOSI-derived metrics and body fat measurements. For example, Figs. 4(a), 4(e), and 4(i) show correlations between adult body fat percentage from skinfold measurements and lipid concentration from DOSI measurements. The Pearson correlation coefficient was 0.7 (p<0.001), 0.7 (p<0.001), and 0.5 (p<0.001) for the femur, humerus, and tibia, respectively. Here the p-value indicates the probability of an uncorrelated system producing a Pearson correlation at least as extreme as the one computed here.^27^

For volunteers <18-year old with skinfold measurements (n=5), correlations between the skinfold percentile and DOSI lipid measurements were not statistically significant. The Pearson correlation coefficient was 0.7 (p=0.190), 0.9 (p=0.221), and 0.5 (p=0.381) for the femur, humerus, and tibia, respectively.

Significant correlations were also found between DOSI lipids and tissue oxygen saturation, total hemoglobin, oxyhemoglobin, and deoxyhemoglin concentrations. The correlation coefficients for tissue oxygen saturation and lipids were −0.7 (p<0.001), −0.7 (p<0.001), and −0.3 (p=0.041) for the femur, humerus, and tibia, respectively, [Figs. 4(b), 4(f), and 4(j)]. The correlation coefficients for THC and lipids were −0.9, −0.9, and −0.8 [all p<0.001, Figs. 4(c), 4(g), and 4(k)]. The correlation coefficients between oxyhemoglobin and lipids were −0.9, −0.8, and −0.7 (all p<0.001). The correlation coefficients between deoxyhemoglobin and lipids were −0.8, −0.9, and −0.7 (all p<0.001).

Figures 4(d), 4(h), and 4(l) show correlations of total hemoglobin with age, none of which were significant. The only statistically significant correlation between age and a DOSI-derived parameter was age versus scattering parameter b at the femur (ρ=0.3, p=0.031, and data not shown).

**Fig. 5:**
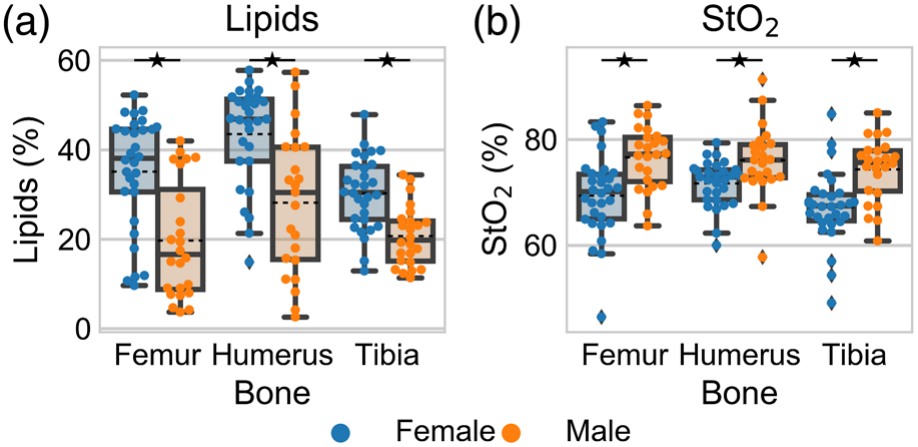
Distribution of (a) lipids and (b) tissue oxygen saturation in the humerus, femur, and tibia for females (blue) and males (orange). Each point is the average of left and right measurements for an individual. The box plots shows the mean (dashed black line), median (solid black line), interquartile range (box), and whiskers extending to 1.5 times the quartiles (thin black line). ⋆ represents a statistical significance between females and males at p=0.05

There were significant and substantial differences in DOSI-derived metrics between the sexes (Fig. 5 and Table S1 in the Supplementary Material). Females had a statistically higher lipid concentration and lower tissue oxygen saturation at each bony location. There were also statistical differences between males and females in the scattering parameter b, oxyhemoglobin, and THC (Fig. S1 in the Supplementary Material). Deoxyhemoglobin had statistical differences between males and females at the femur and humerus, water concentration had statistical differences in the humerus and tibia, and the scattering parameter a had a difference in the humerus.

A comprehensive body scan of a single individual is shown in the Supplementary Material (Figs. S2–S4).

### 3.4 Quantifications of Variation with Regression Analysis

An ordinary least squares model was used to identify significant-independent variables for each DOSI-derived metric. The coefficients from significant variables (p<0.05) are shown in Table 3 in which each column represents the results for the model corresponding to a specific DOSI-derived metric. The most influential independent variable for each DOSI metric is bolded. Body fat percentage was a statistically significant-independent variable for all DOSI metrics. Age and the interaction term between sex and body fat percentage were not statistically significant variables.

**Table 3:** Coefficients from statistically significant-independent variables (p<0.05) in ordinary least squares models for each DOSI metric. The most influential independent variable for each DOSI metric is bolded. Insignificant variables are empty. Note that for significant variables (e.g. Bone), the coefficients for all categories of that variable are shown even if a particular category (e.g. tibia) is not significant

Independent variables	DOSI-derived metrics							
	a	b	${\mathrm{HbO}}_{2}$	HHb	Water	Lipids	THC	${\mathrm{StO}}_{2}$
$R_{\mathrm{adj}}^{2}$	0.37	0.53	0.53	0.36	0.59	0.61	0.55	0.42
Intercept	0.8	−0.9	55.0	14.6	23.1	14.2	69.7	76.7
Body fat	<0.01	<0.01	−1.1	−0.1	−0.3	**0.8**	−1.2	−0.3
Bone (humerus)	−0.1	**0.2**	2.8†		−3.2	7.9	2.9†	1.7†
Bone (tibia)	−0.1	−0.3	−6.4		**11.9**	−2.5†	−6.7	−2.4
Sex (male)		−0.3	16.2	2.1		−11.9	18.3	**5.8**
Race (Black)		−0.1†	−2.9†	−0.5†		1.5†	−3.3†	−0.7†
Race (Multiracial)		0.2†	−31.1	−5.3		18.8	−36.4	−14.5
Race (other)		0.2†	−14.1	−2.5		9.7	−16.7	−2.5†
Race (unknown)		0.2†	−2.9†	−0.2†		−4.6†	−3.0†	−2.2†
Race (White)		0.2	−1.4†	−0.6†		−0.7†	−2.0†	−0.4†
Ethnicity (non-Hispanic)	−0.1							
Age								
Body fat*sex								

† p>0.05.

Sex was a statistically significant variable for all DOSI metrics except for scattering parameter a and water. Ethnicity was statistically significant for scattering parameter a. Bony location was a statistically significant variable for all DOSI metrics except deoxyhemoglobin; however, not all anatomic locations were statistically significant (e.g., oxyhemoglobin, lipids, THC, and tissue oxygen saturation). Race was a statistically significant variable for scattering parameter b, oxyhemoglobin, deoxyhemoglobin, lipids, THC, and tissue oxygen saturation.

The largest amount of variance in the model was explained by lipids ($R_{\mathrm{adj}}^{2}=0.61$), and the least was explained by the a parameter ($R_{\mathrm{adj}}^{2}=0.37$). Bony location was the most influential variable for scattering parameters a and b and water. Sex was the most influential variable for tissue oxygen saturation. Body fat percentage was the most influential variable for oxyhemoglobin, deoxyhemoglobin, THC, and lipids.

## 4 Discussion

This study presents the first comprehensive diffuse optical measurements of the distal humerus, distal femur, and proximal tibia in healthy volunteers. These three anatomic locations are the primary site for 66% of osteosarcomas.^16^ Key findings include differences in only some DOSI parameters at the three anatomic sites, strong correlations between body fat and hemoglobin, strong sex differences in DOSI parameters, a lack of age-related correlations with DOSI parameters, and preliminary evidence that race and ethnicity may effect DOSI measurements. These results are now discussed in the context of prior work.

### 4.1 Optical Parameters in Different Anatomic Regions

The largest differences between the bony locations measured in this study occurred in the a and b scattering parameters, water, and lipids. These differences may be due to the underlying differences in the structure of the bony locations. The femur and tibia locations were at the ends of weight-bearing bones, representing a larger share of trabecular bone, whereas the humerus measurement was on the shaft, representing more cortical bone and bone marrow. The humerus had a lower a and higher b values compared to the femur and tibia, as well as lower water and higher lipids. The scattering parameter a is related to the number of scatterers and b is related to the particle size distribution.^28^ The differences in scattering may be due to the differences in bone architecture including the more porous nature of trabecular bone compared to cortical bone. The higher share of lipids may be due to the higher lipids present in bone marrow on the humerus shaft measurement as compared to trabecular bone measured in the femur and tibia.^29^

A surprising finding of this study was that no statistical differences were observed in hemoglobin concentrations between bony locations. This differs from some prior reports that demonstrated hemoglobin differences between bony locations.^13^^,^^17^ For example, Sekar et al.^17^^,^^30^ used time-resolved diffuse optical spectroscopy to measure the proximal femur (trochanter), distal and proximal radius, distal and proximal ulna, and calcaneous. They found statistically significant differences in oxyhemoglobin, deoxyhemolgobin, and oxygen saturation between many of these sites. The lack of differences in hemoglobin concentrations between anatomic sites measured in this study may be due to the fact that these three regions had a similar blood supply and metabolic activity; other possibilities are discussed below.

When comparing the absolute values of DOSI parameters to prior work, several differences were found with prior literature. For example, the lipid concentration and tissue oxygen saturation reported by Sekar et al. were higher, and the oxyhemoglobin, deoxyhemoglobin, and THC were generally lower than the results reported here. The scattering parameters a and b were similar. The oxygen saturation values reported by Farzam et al.^12^ for the manubrium were similar to those reported here, but the total hemoglobin values were generally higher by at least 20  μM. The oxygen saturation, lipids, and water concentrations reported by Pifferi et al.^11^ in the calcaneous were similar to this study, but total hemoglobin was substantially lower. Thus far, the only known frequency-domain DOS bony measurement of healthy volunteers has been in the patella^31^ and the tibia in our prior work.^32^ The chromophore values for oxyhemoglobin and deoxyhemoglobin reported for the patella were lower than the mean concentrations measured in the three anatomic sites in this study. The assumption of 15% water concentration in the patella^31^ is similar to water concentrations measured in this study for the femur (16.0%) and humerus (12.5%), but not the tibia (27.8%). Differences in chromophore concentrations with prior work could simply be that different bony sites were measured in this study. The closest match between prior studies and this study were the proximal femur (Sekar et al.) and the distal femur (this study). Even in this case, oxy- and deoxyhemoglobin concentrations were quite different, and the oxygen saturation reported for the proximal femur was at least 10% lower than the distal femur measurements here.

Another possible explanation for the differences observed in this study compared to prior studies is a partial volume effect in which our instrument measured a different portion of bone versus overlying tissue compared with prior works. For example, the lack of differences in hemoglobin between the anatomic sites could be due to a partial volume effect in which the superficial lipid layer obscures underlying differences in vasculature in the bony regions. Additionally, the partial volume effect could be a reason why the tibia had the highest water concentration: water is one of the top three components of bone^33^ and the tibia is the most superficial of the bones measured here. Additionally, the humerus had the lowest water and highest lipid concentration, which could be due to the greater subcutaneous lipid layer over this region, or it could be from measuring over the bone shaft where the fatty bone marrow is stored. Since a similar source–detector separation was used by this study and some prior studies (e.g., 28 mm versus 25 mm for this study and Sekar et al. 2016, respectively), it may also be that the regions measured in this study had thicker overlying tissue. Tissue thicknesses were not explicitly measured in this study.

### 4.2 Correlations Between Parameters

There were significant correlations between DOSI lipid concentrations and adult body fat percentage, with the femur and humerus exhibiting the strongest correlations (ρ=0.7 for both). More strikingly, this study showed strong correlations between DOSI lipid measurements and hemoglobin measurements. These strong negative correlations (ρ=−0.8 for tibia, ρ=−0.9 for femur and humerus) between THC and DOSI lipids in this study agrees with prior DOS studies that have shown negative correlations between THC and body mass index (BMI)^12^ or fat mass.^10^ This correlation does not appear to be from crosstalk between hemoglobin and lipids due to instrument bias or noise. Nonimaging studies have also observed a negative correlation between BMI and hemoglobin.^34^^,^^35^ Given that the blood volume to body weight ratio is lower for obese and overweight individuals than underweight and normal weight individuals, the negative correlation is perhaps unsurprising.^36^^,^^37^ Biological reasons for the correlation could relate to reduced blood flow, capillary density, or blood perfusion.^38^

### 4.3 Sex

Substantial sex differences were observed in this study. Females generally had higher lipid concentrations and b scattering values and lower hemoglobin, oxygen saturation, and a scattering values. There have been few prior reports of sex differences at bony anatomic regions, but Farzam et al.^12^ reported that tissue oxygen saturation was 2% lower in females compared to males at the manubrium. In this study, females on average had a 6.3% lower oxygen saturation compared to males. The same study found no differences between the sexes in THC at the manubrium, which differs from this study in which females had almost 20  μM less total hemoglobin compared to males. Discrepancies between these studies could stem from the location of the measurement or the distribution of body fat percentage or BMI of the volunteers. A notable difference between the studies is that the distribution of BMI for men and women were quite different, with women having a lower BMI than men in Farzam et al.’s study.

In this study, the body fat percentage distribution was statistically different between males and females; the average body fat percentage was 26.7% for adult females and 18.2% for adult males. This difference is expected since females and males store fat differently.^39^^,^^40^ However, it is of note that the males and females in this study had a lower body fat percentage compared to a representative sample of the US population.^41^

Sex differences have not been extensively explored using diffuse optical technologies, and many prior DOSI clinical studies have focused on breast cancer, which predominately affects women.^6^^–^^9^ These results make it clear that sex differences are substantial in these anatomic locations and should be taken into account when analyzing mixed-sex data sets or when developing diagnostic or prognostic thresholds in pathological conditions affecting these sites.

### 4.4 Age

Another surprising result of this study was the lack of correlation between DOSI parameters and age. Despite the fact that a very wide range of ages was included in this study (4 to 72 years), age and the scattering parameter b on the femur were the only statistically significant correlation (data not shown ρ=0.3, p=0.031), and this correlation was not significant when accounting for multiple comparisons. Age was also not a statistically significant variable in any of the regression analyses. This results agree with Farzam et al., which showed that age was not an influential parameter in describing variation in the manubrium of adults aged 25 to 40.^12^ However, in a different DOS study of the calcaneous of seven female adults aged 26 to 82, THC increased and the scattering a parameter decreased with age.^11^ Prior work has shown that THC measured from blood draws increase with age until age 20 to 23 years, suggesting that a correlation between hemoglobin concentration and age might be expected.^42^ However, the large variation within chromophore values and the small sample size of minors suggest that more data may be needed to fully evaluate age related differences.

### 4.5 Race and Ethnicity

The inclusion of race and ethnicity as variables in the regression analyses improved $R_{\mathrm{adj}}^{2}$ for all DOSI-derived metrics except water. The greatest increases in $R_{\mathrm{adj}}^{2}$ were 0.07 for a and THC, with an average increase of 0.05 (data not shown). Large cohort studies have established statically different hemoglobin concentrations between racial and ethnic groups,^43^^–^^45^ though there is debate if these differences have clinical implications.^46^^–^^48^ This study is underpowered to detect hemoglobin differences between racial and ethnic groups, but suggests that these are likely important variables needed to explain the variation in DOSI measurements.

### 4.6 Implications for Osteosarcoma and other Bone Pathologies

We have previously reported optical properties and chomophore values of six patients with sarcoma measured with DOSI.^13^ Five of these six patients were measured at an anatomic location also measured in this work. In general, the chromophore values of tumor and normal tissue measured from the sarcoma patients were similar to the chromophore values of volunteers measured in this study, an exception being that all sarcoma patients had a higher lipid concentration. We note that one of the six sarcoma patients was excluded from analysis in our prior because of low SNR, an issue that was not present in any of the 55 volunteers in this study. This suggests that patients with sarcoma may be more challenging to measure with DOSI compared to healthy volunteers, likely due to the the accumulation of hemoglobin in hypoxic and necrotic tumors. Both studies were conducted with the same handheld probe and with the same source–detector separation, but with different back-end electronics.

This study helps to set the baseline for future studies of sarcomas including osteosarcoma and Ewing’s Sarcoma and may be useful for other bone pathologies such osteoporosis. The large chormophore variation measured within the population supports the practice of using contralateral normal tissue as a control in clinical studies^9^^,^^13^ and presents opportunities for DOSI in applications of diseases with an asymmetry in the body (e.g., cancer and nonosteoporotic fragility fractures^2^).

### 4.7 Limitations

There were several important limitations of this study. One potential limitation is the choice of chromophores. In this study, only oxyhemoglobin, deoxyhemoglobin, water, and lipids were fit to measured broadband absorption spectra. In contrast, Sekar et al.^17^^,^^30^ included collagen and a background variable as chromophores and Farzam et al.^31^ assumed a 15% water concentration. Myoglobin is also a likely contributor to absorption in muscular and bony tissues. Myoglobin and hemoglobin are similar spectrally and likely cannot be accurately separated in this context.^49^ Collagen was not included in part because this study only took measurements up to 1000 nm, and collagen extractions have been shown to benefit from longer wavelengths, which include the absorption peaks at 1025 and 1200 nm.^17^^,^^50^^–^^52^ Additionally, there are limited experimental methods to validate the accuracy of collagen fits. It is likely that if collagen was included as a chromophore, the lipid, water, and deoxyhemoglobin concentrations reported here would decrease to some extent.^51^

Another potential limitation is that the bone depth and underlying tissue structure is unknown for the volunteers. Although measurement points are aligned to bones and bony landmarks, measurements on the humerus and the tibia were taken at an absolute distance from a bony landmark on all volunteers. Differences in height and bone length could cause measurement locations to be on different parts of the bone or on different vascular beds.^2^ Bony locations were chosen to minimize the effect of overlying muscle, however, there may be minimal muscle contribution at the humerus, which is seen in a comparison of body symmetry (Table S2 in the Supplementary Material).

The light propagation model used to process the data is a potential limitation of this study. The P1 diffusion approximation of the Boltzmann transport equation and its boundary conditions assume a homogeneous, optically diffusive, and semi-infinite media.^5^^,^^19^ However, *in vivo* measurements of bone involve multiple chromophores, layered tissue, and complex microarchitecture. This light propagation model has not been validated for *in vivo* measurements of bone, which is porous and highly structured. It is notable that previous studies of NIR absorption and reduced scattering spectra of bone indicate that bone is an optically diffusive media^11^ and that light propagation in trabecular bone can be analyzed using the diffusion approximation.^53^

The handheld probe used in this study was also a limitation. The probe had a relatively large $6.6\times 8.9\;{\mathrm{cm}}^{2}$ footprint, which prevented measurement contact in some angular anatomic sites. Individuals with lower body fat percentage and higher muscle definition added additional curvature to already angular locations and bony protuberances became more prominent. Technological development to the probe design would facilitate improved measurements at these anatomic locations.

The distribution of enrolled volunteers was also a potential limitation in this study. Bone cancers predominantly affect children, however, minors only represented 12% of the enrolled volunteers. More pediatric volunteers are likely needed to confirm if there are any age-related effects. Similarly, larger sample sizes are needed to quantify any ethnic- and race-related effects since the majority of participants were White (49%) and not Hispanic or Latino (87%). When all regression parameters are included, the largest $R_{\mathrm{adj}}^{2}$ value for any chromophore or scattering parameter is 0.61, indicating there is still a substantial variation not explained by anatomic location, age, sex, body fat percentage, race, or ethnicity.

### 4.8 Conclusions

In summary, this study found differences in optical scattering, water, and lipids between three bony anatomic locations, but no statistical differences in hemoglobin-based parameters. Strong correlations were found between body fat estimates determined by skin fold calipers and DOSI lipids. Additionally, strong correlations occurred between DOSI lipids and hemoglobin-based parameters. Correlations between DOSI lipids and hemoglobin were also observed in the single-subject body scan. Strong sex differences were observed in DOSI lipids and hemoglobin-based parameters. No age-related correlations were observed in this data set, and no statistical differences were observed between pediatric and adult populations. Race and ethnicity were significant variables for some regression models. In the future, DOSI may provide a new means to diagnose and track treatment of bone pathologies.
