# Supplementary material for: Characterization of bony anatomic regions in pediatric and adult healthy volunteers using diffuse optical spectroscopic imaging
Source: J Biomed Opt. 2020 Aug 12;25(8):086002. doi: 10.1117/1.JBO.25.8.086002 (PMC7422854; doi:10.1117/1.JBO.25.8.086002)
Supplement: Supplementary file 1 [file JBO_025_086002_SD010.pdf]

Supplementary Material

S1 Sex Differences

The mean and standard deviation of oxyhemoglobin, deoxyhemoglobin, water, lipids, total hemoglobin, and tissue oxygen saturation are reported for males and females for each bone in Table S1 and Figure S1.

Table S1 Mean and standard deviation for optical properties and chromophore concentrations in bones for males and females.

Variable	Sex	Femur	Humerus	Tibia
a (mm^{-1})	Female	0.8 ± 0.1	0.6 ± 0.1	0.7 ± 0.1
a (mm^{-1})	Male	0.8 ± 0.1	0.7 ± 0.1	0.7 ± 0.1
b (AU)	Female	-0.5 ± 0.2	-0.4 ± 0.1	-0.8 ± 0.2
b (AU)	Male	-0.9 ± 0.4	-0.6 ± 0.4	-1.2 ± 0.3
HbO_2 (μM)	Female	26.9 ± 14.9	28.2 ± 11.0	22.9 ± 8.5
HbO_2 (μM)	Male	45.9 ± 19.6	48.2 ± 27.7	36.3 ± 9.2
HHb (μM)	Female	10.4 ± 3.0	10.5 ± 3.0	10.4 ± 1.9
HHb (μM)	Male	12.4 ± 3.5	12.8 ± 3.2	11.7 ± 2.8
Water (%)	Female	14.7 ± 3.1	11.6 ± 1.7	25.2 ± 8.2
Water (%)	Male	17.6 ± 6.4	13.7 ± 3.8	31 ± 9.5
Lipids (%)	Female	35.1 ± 12.7	43.5 ± 11.1	30.3 ± 8.1
Lipids (%)	Male	21.2 ± 14.5	28.7 ± 15.6	21.1 ± 6.6
THC (μM)	Female	37.3 ± 17.2	38.7 ± 13.8	33.3 ± 9.5
THC (μM)	Male	58.3 ± 22.2	61.0 ± 29.6	47.9 ± 10.6
StO_2 (%)	Female	69.3 ± 7.7	71.7 ± 4.3	67.2 ± 6.8
StO_2 (%)	Male	77.2 ± 5.9	76.3 ± 6.6	74.6 ± 5.9

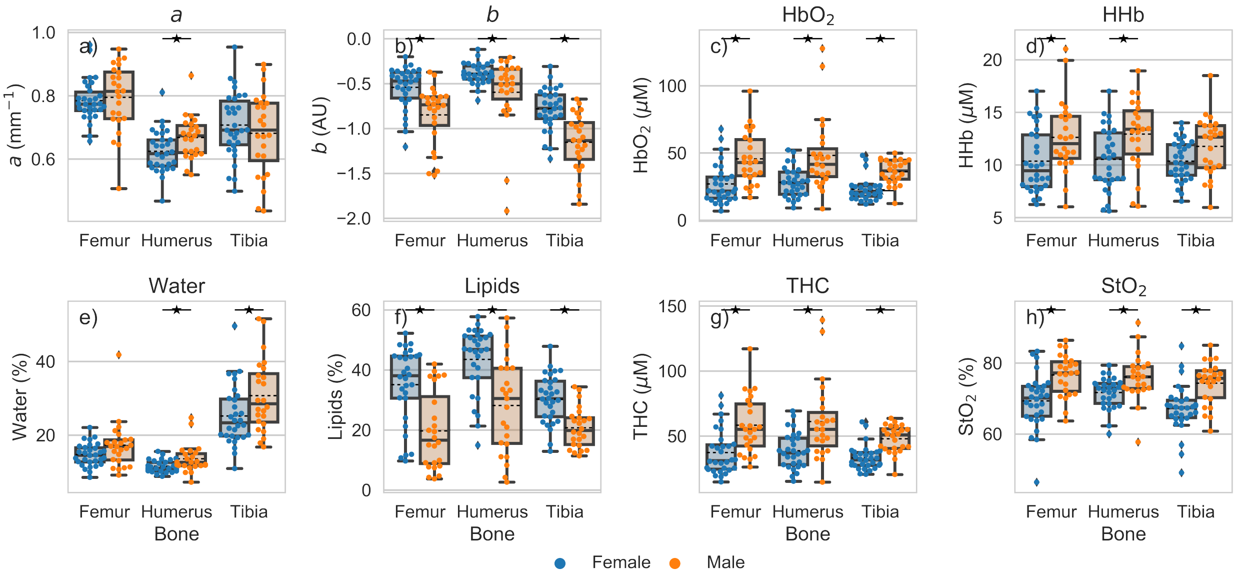

Fig S1 Distribution of optical property parameters (a) a and (b) b and concentrations of (c) oxyhemoglobin, (d) deoxyhemoglobin, (e) water, (f) lipids, (g) total hemoglobin, and (h) tissue oxygen saturation for the humerus, femur, and tibia for females (blue) and males (orange). Each point is the average of left and right measurements for an individual. The box plot shows the mean (dashed black line), median (solid black line), interquartile range (box), and whiskers extending to 1.5 times the quartiles (thin black line). $\star$ represents a statistical significance between females and males at p = 0.05. Panels (f) and (h) are also shown in Fig 5.

S2 Body Symmetry

Optical properties and chrmophore concentrations on left and right sides of the body were compared for each bone by taking the ratio of average left to right measurements. The mean and standard deviation for the ratio of oxyhemoglobin, deoxyhemoglobin, water, lipids, total hemoglobin, and tissue oxygen saturation are reported for each bone in Table S2. A one sample T-test was used to compare the distribution of ratios to 1 and no multiple comparison test was used.

Table S2 Mean and standard deviation for the ratio of left to right measurements of optical properties and chromophore concentrations in bones.

Variable	Femur (n=45)	Humerus (n=38)	Tibia (n=53)
a (mm^{-1})	1.0 ± 0.1	0.8 ± 0.1	1.0 ± 0.2
b (AU)	1.0 ± 0.3	0.7 ± 0.3	0.9 ± 0.2
HbO_2 (μM)	1.1 ± 0.3	1.2 ± 0.5	1.0 ± 0.2
Hb (μM)	1.1 ± 0.2	1.1 ± 0.3	1.1 ± 0.2
Water (%)	1.1 ± 0.2	0.8 ± 0.2	1.0 ± 0.3
Lipids (%)	1.1 ± 0.4	1.4 ± 0.6	1.1 ± 0.3
THC (μM)	1.0 ± 0.3	1.1 ± 0.4	1.0 ± 0.2
StO_2 (%)	1.0 ± 0.1	1.0 ± 0.1	1.0 ± 0.1

At the femur, the a parameter ratio was the only DOSI-derived metric to statistically differ from 1 ($p = 0.004$). The tibia had ratios statistically differ from 1 for the b parameter ($p = 0.013$) and deoxyhemoglobin ($p = 0.042$). The ratios for the a and b parameters (both $p < 0.001$), deoxyhemoglobin ($p = 0.034$), water ($p < 0.001$), and lipids ($p < 0.001$) all statistically differed from 1 at the humerus.

Generally speaking, the boniest locations had the most symmetry. Arm usage and muscle contribution at the humerus may contribute to the asymmetry. Handedness and footedness was not recorded for volunteers.

S3 DOSI Body Scan

As a proof of concept to explore chromophore variation across the body, a volunteer participated in a comprehensive body scan. A 6 by 6 cm square grid was transferred onto the skin surface using a transparency and nonpermanent surgical marker. The grid spanned from the center of

manubrium across the clavical to the proximal humeral head and down the arm to the distal ends of radius and ulna and down the ribs, hip, and thigh to foot. Grid locations are shown in Figure S2.

The strict quality control protocols for light leakage from Section 2.5 were not used for this measurement. All measurements were processed using the methods described in Section 2.4. Chromophore values were displayed as 2D maps and the Pearson's correlation coefficient was used to quantify the relationship between DOSI metrics at a given measurement location.

Volunteer 3367-046, a 27 year old white non-Hispanic female with 27.8% body fat percentage, completed a comprehensive body DOSI scan. A total of 149 DOSI measurements were taken, of which 133 (89%) passed the quality control procedure outlined in Section S3.

Figure S3 shows the spatial distribution of lipids and tissue oxygen saturation in this volunteer. Lipids varied from 9.4% to 63.7% with an average of 41.5%. Tissue oxygen saturation was lowest on the foot (50.6%) and highest on the torso (88.1%). The mean tissue oxygen saturation for the body was 73.4%.

The correlation between several DOSI metrics are shown in Figure S4. The correlation coefficient for tissue oxygen saturation and lipids was 0.3 ($p < 0.001$) and the correlation coefficient for total hemoglobin concentration and lipids was -0.9 ($p < 0.001$).

The DOSI comprehensive body scan was collected from only a single volunteer due to the effort and time needed to acquire this data. While generalizations cannot be made based on this single subject, the spatial variation of chromophores provides some insight to lipid, water, and hemoglobin distributions in the body. There was generally lower lipids and oxygen saturation at the periphery (arms and lower legs) compared with the torso and upper legs. Additionally, there was a strong negative correlation between DOSI lipids and total hemoglobin ($\rho = -0.9$), which was similar to the correlation observed over the population. Curiously, a positive correlation between tissue

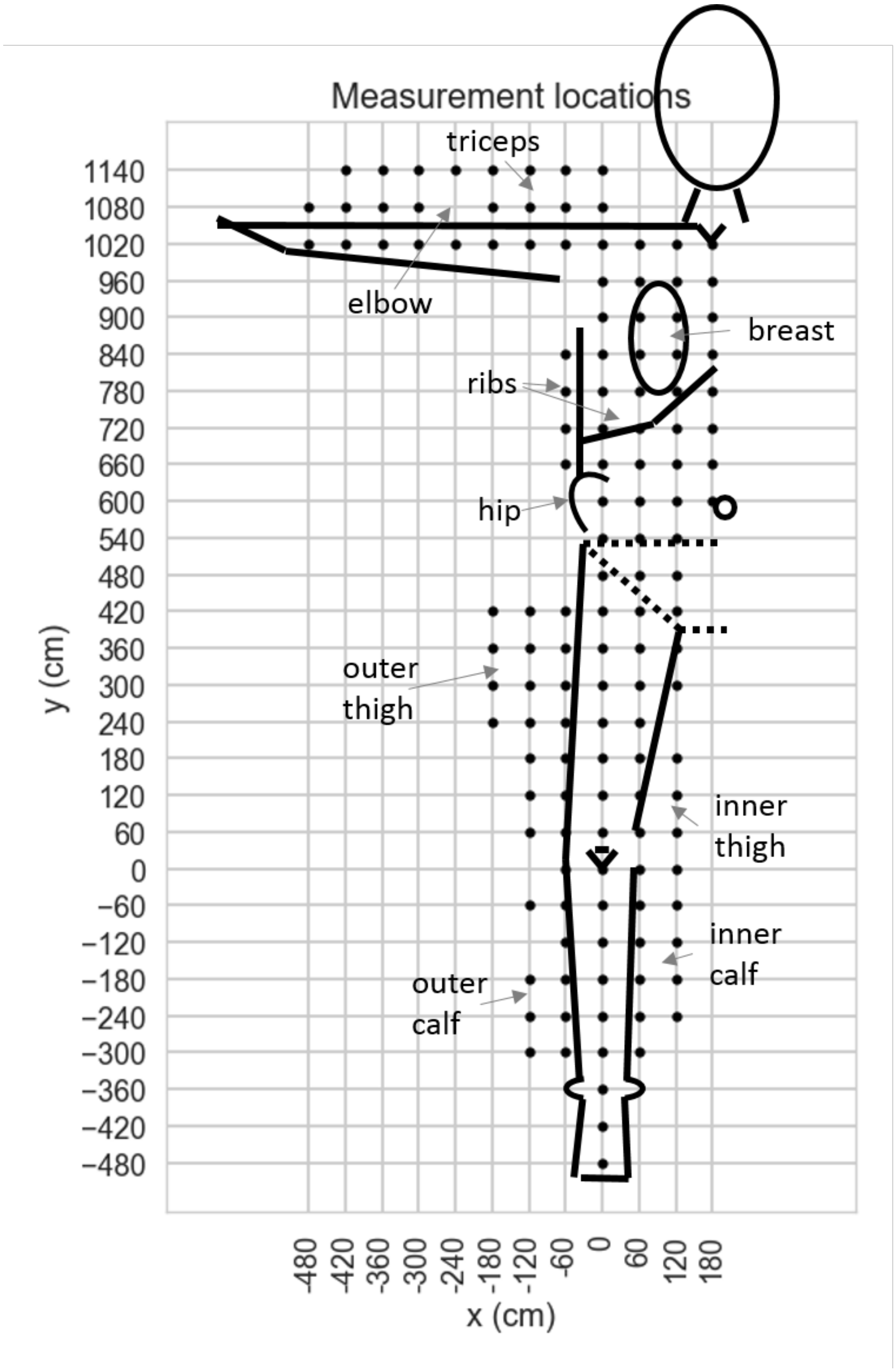

Fig S2 Schematic of measurement locations for the body scan. Each point represents a measurement and arrows point to anatomic features.

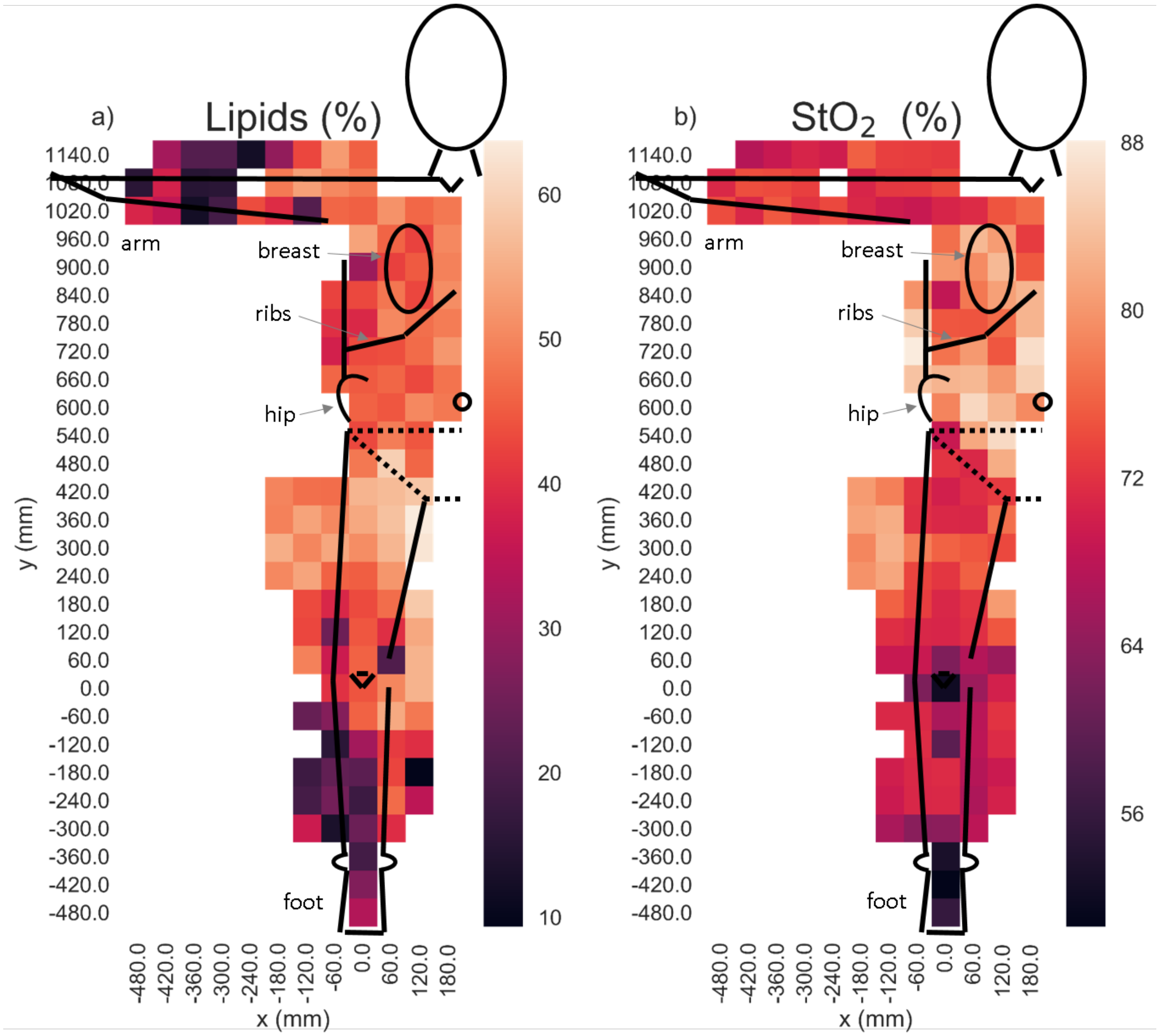

Fig S3 Spatial Distribution of (a) lipids and (b) tissue oxygen saturation for a 27 year old white non-Hispanic female with 27.8% body fat percentage. Schematic of the body is overlaid for orientation.

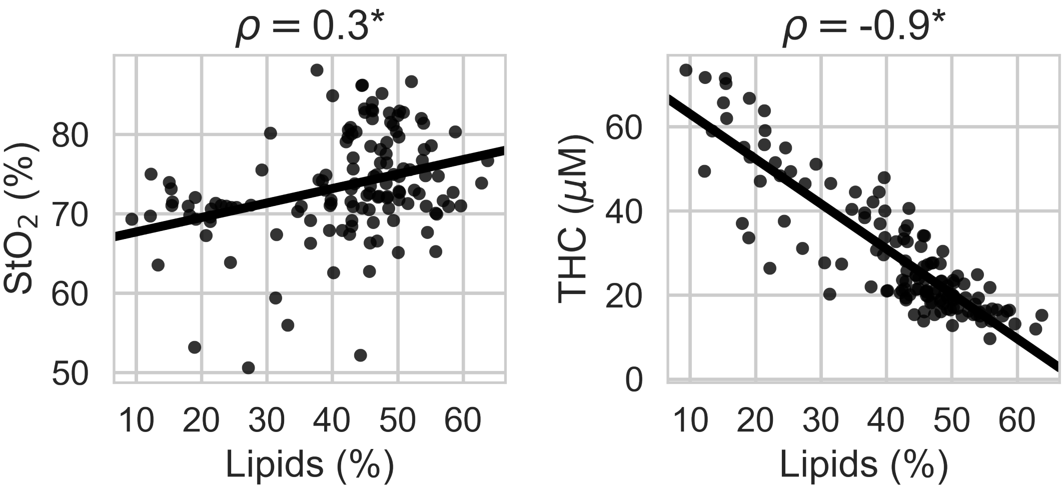

Fig S4 Correlation of lipids and tissue oxygen saturation (a) and lipids and total hemoglobin concentration (b) within the body for a 27 year old white non-Hispanic female with 27.8% body fat percentage. ⋆ represents statistical significance at a significance level of p = 0.05

oxygen saturation and lipids was observed in the individual (ρ = 0.3) while a negative correlation was observed in the population (ρ = -0.3, -0.7, and -0.7 for tibia, femur, and humerus respectively). As DOSI instrumentation continues to improve in acquisition speed and the ability to scan over larger spatial areas, more analyses of intra-subject measurements may lead to new insights to human pathophysiology.
